# Taxonomy Informed Clustering, an Optimized Method for Purer and More Informative Clusters in Diversity Analysis and Microbiome Profiling

**DOI:** 10.3389/fbinf.2022.864597

**Published:** 2022-04-27

**Authors:** Antonios Kioukis, Mohsen Pourjam, Klaus Neuhaus, Ilias Lagkouvardos

**Affiliations:** ^1^ Medical School, University of Crete, Heraklion, Greece; ^2^ Core Facility Microbiome, ZIEL – Institute for Food & Health, Technical University Munich, Freising, Germany; ^3^ Institute of Marine Biology, Biotechnology and Aquaculture, Hellenic Centre for Marine Research, Heraklion, Greece

**Keywords:** taxonomic classification, microbial diversity, clustering, microbiome analysis, amplicon sequencing, NGS processing pipeline

## Abstract

Bacterial diversity is often analyzed using 16S rRNA gene amplicon sequencing. Commonly, sequences are clustered based on similarity cutoffs to obtain groups reflecting molecular species, genera, or families. Due to the amount of the generated sequencing data, greedy algorithms are preferred for their time efficiency. Such algorithms rely only on pairwise sequence similarities. Thus, sometimes sequences with diverse phylogenetic background are clustered together. In contrast, taxonomic classifiers use position specific taxonomic information in assigning a probable taxonomy to a given sequence. Here we introduce Taxonomy Informed Clustering (TIC), a novel approach that utilizes classifier-assigned taxonomy to restrict clustering to only those sequences that share the same taxonomic path. Based on this concept, we offer a complete and automated pipeline for processing of 16S rRNA amplicon datasets in diversity analyses. First, raw reads are processed to form denoised amplicons. Next, the denoised amplicons are taxonomically classified. Finally, the TIC algorithm progressively assigning clusters at molecular species, genus and family levels. TIC outperforms greedy clustering algorithms like USEARCH and VSEARCH in terms of clusters’ purity and entropy, when using data from the Living Tree Project as test samples. Furthermore, we applied TIC on a dataset containing all *Bifidobacteriaceae*-classified sequences from the IMNGS database. Here, TIC identified evidence for 1000s of novel molecular genera and species. These results highlight the straightforward application of the TIC pipeline and superior results compared to former methods in diversity studies. The pipeline is freely available at: https://github.com/Lagkouvardos/TIC.

## 1 Introduction

Today, profiling of microbial communities is often conducted by inexpensive and high throughput DNA-sequencing (i.e., next generation sequencing, NGS). These profiling techniques often rely on amplifying target marker genes by using the polymerase chain reaction (PCR) and subsequent parallel sequencing (Nocker et al., 2007). The obtained sequences are then compared to gene databases for probable taxonomic assignment. All assigned sequences of a sample result in a microbial profile. Since many years, the 16S rRNA gene is the primary target for most microbiome and diversity studies due to its versatility and phylogenetic information density (Woese et al., 1980). This technique can even resolve the microbial profile down to strain level, as shown in a study of Johnson et al. (2019).

In common approaches, sequence reads are usually *de novo* clustered into groups based on their sequence similarity (Blaxter et al., 2005), (Porter and Hajibabaei, 2018). Subsequently, the centroids of these similarity groups are classified to the closest known taxonomic level, obtaining so called Operational Taxonomic Units (OTUs). To form these clustered groups, multiple methods have been proposed. Several are based on calculating pairwise sequence similarities from multiple sequence alignments using UPGMA or neighbor-joining algorithms (Liu et al., 2009), (Li, 2015). However, these algorithms are computationally demanding processes and not the fastest in finding similar sequences in multiple sequence alignments, especially when using large similarity matrices as needed in microbiome studies. Thus, heuristic distance-based greedy clustering (DGC) and abundance-based greedy clustering (AGC) algorithms have been developed that produce the required clustering with a single pass through the data and are much faster (Edgar, 2010), (Rognes et al., 2016). Taken together, compromises must be taken between accurate and thorough methods on one side and fast analysis methods on the other side. The shortcomings of the DGC and AGC algorithms follow from their single pass through the data. For instance, these algorithms choose the first amplicon from the sequence pool and take it as the first OTU centroid. The next sequence is compared to the first based solely on similarity. If sufficiently similar, the sequence is added to the centroid. In case the sequence is too different, a second centroid (second OTU) is initiated. Thus, an OTU is formed by adding sequences being similar to the centroid above a defined threshold. This step is repeated with the remaining, not yet clustered sequences until all are assigned to OTUs (Edgar, 2010), (Rognes et al., 2016). Hence, the order of sequences in each data set strongly influences the resulting clustering output. The sequential addition of new sequences to existing OTUs might even sort sequences into different OTUs even though they have a significant similarity. However, these sequences are never evaluated together due to the sequential nature of the process. Ultimately, this causes random variation in microbial community assignments (Koeppel and Wu, 2013). While preordering the sequences based on their abundance in the dataset increases the reproducibility of the clustering process (Edgar, 2013) this does not eliminate the possible misplacements of sequences in different OTUs (Edgar, 2013).

More recent approaches argue against the process of clustering and rather support the processing of sequences only by removal of chimeras and sequencing errors down to what is referred to as denoised sequences. Two algorithms are the most common used for denoising, DADA2 (Callahan et al., 2016) and UNOISE3 (Edgar, 2016). The results are, as said, denoised sequences in both cases, while the creators of DADA2 call their result amplicon sequence variant (ASV) and the author of USEARCH names them zero-radius OTU (zOTU).

In any case, after having processed all sequences to a list of OTUs representatives or denoised sequences they are classified to their closest taxonomy possible. The outcome of this process is dependent on initial primer choice (i.e., the variable region of the 16S rRNA gene used), the software chosen to perform each task and reference databases used (Abellan-Schneyder et al., 2021), including RDP (Lan et al., 2012) or SILVA (Quast et al., 2012). Unfortunately, reference databases have partially different taxonomic nomenclature, differ in update frequency, and unavoidable errors in such reference databases are affecting the quality and comparability of the results (Sierra et al., 2020). For example, the database GreenGenes DeSantis et al. (2006) has not been updated since 2013 and should not be used anymore. Through the years, SILVA and RDP have distinguished themselves and are currently the most frequently used by classifiers.

**FIGURE 1:**
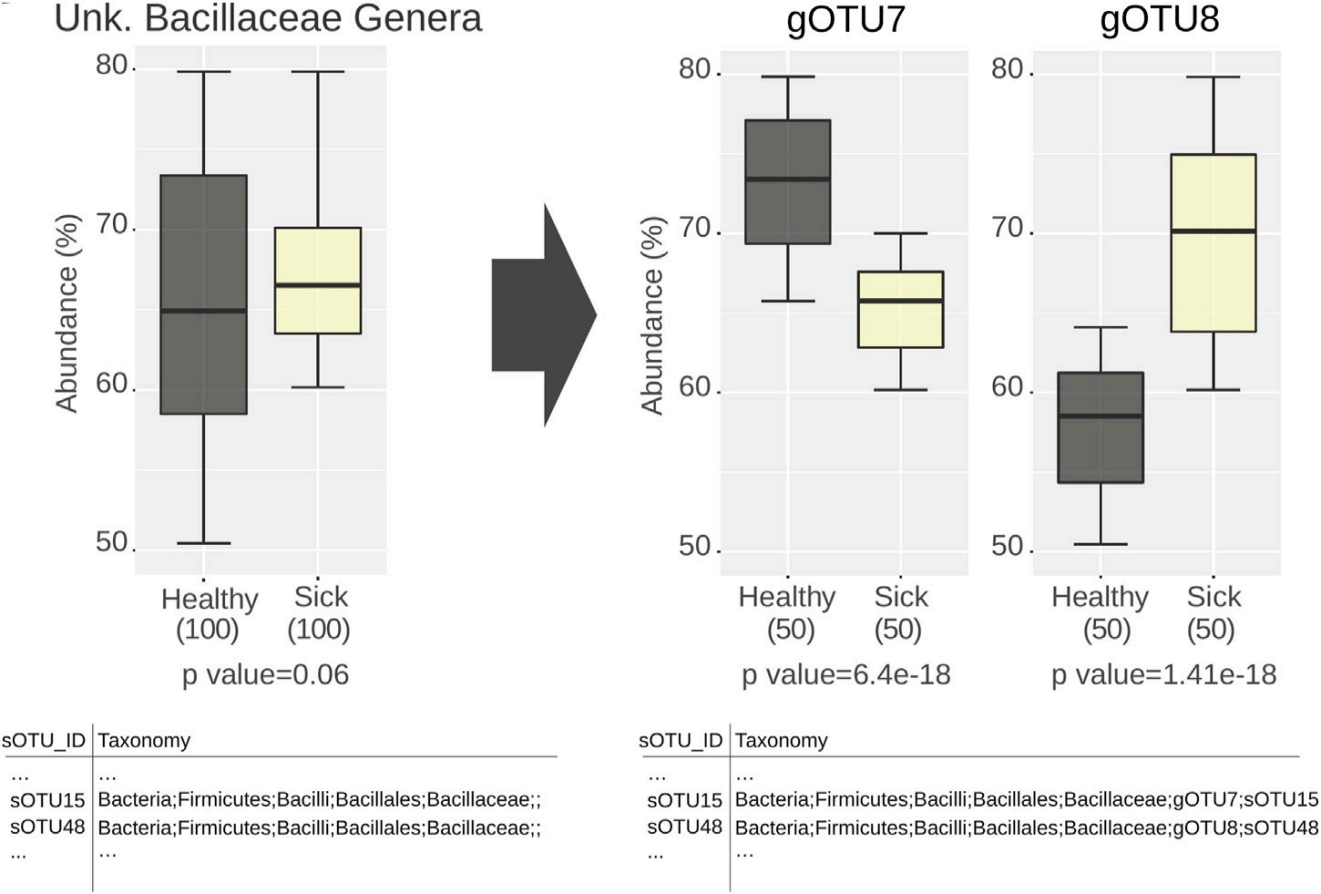
Schematic representation of the shortcomings of missing detailed taxonomic assignments in microbiome analysis. OTUs missing taxonomic classification for a certain level (e.g., genus) are analyzed together under the unknown label. The resulting conclusions can be deceiving when the constituting natural divisions are present nonuniformly across conditions.

Taxonomic classification performs well on sequences from characterized bacteria and archaea, correctly assigning them up to their genus level. However, unknown sequences not represented in the reference databases result in incomplete taxonomic paths. In every sample, there will be sequences from yet undescribed taxa. For instance, in gut samples, the proportion of OTUs that can be assigned to fully described species ranges from 35 to 65%. For environmental samples, this ratio is even lower (Lagkouvardos et al., 2017). For analysis of ecological patterns in higher taxonomic levels (e.g., family), sequences with incomplete taxonomic classification are collectively binned intro groups of “Unknown taxon” or simply discarded. Obviously, these problems limit the resolution of the biological signal that could have been extracted from available sequence data (Figure 1).

**FIGURE 2:**
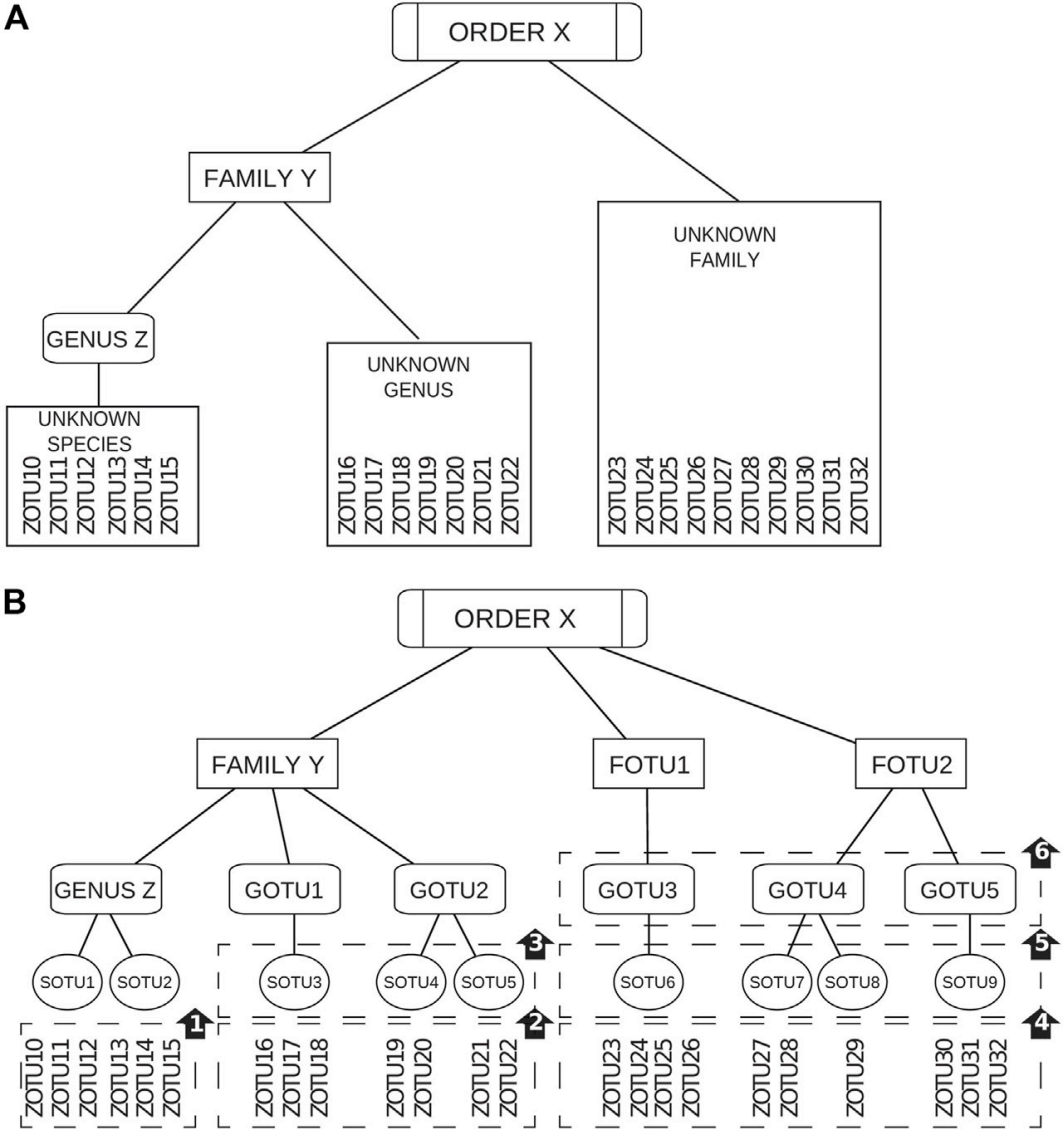
Simplified representation of the TIC algorithm. **(A)** Sequences are divided based on their identified taxonomic level. **(B)** *1*) Denoised sequences within the same genus are clustered to produce sOTUs. *2*) Sequences of unknown genera within the same family are clustered into sOTUs. *3*) Produced sOTUs are further clustered into novel gOTUs. *4*) Sequences of the unknown family are first clustered into sOTUs. *5*) Those sOTUs produce gOTUs. *6*) fOTUs are formed from the gOTUs of an unknown family.

Here we present “Taxonomy Informed Clustering” (TIC), a novel tool that flips the above paradigm, i.e., classifying after clustering. Here, we first taxonomically classify each sequence before any clustering is conducted. The taxonomic information acquired and now attached to each sequence acts both as a guide and as a limit in an incremental clustering process (Figure 2). Thus, the dataset is divided into subsets following the assigned taxonomies and, working within each subset, we avoid merging sequences from diverse lineages together. As a result, the created clusters have a higher purity and their number resembles more that of the intrinsic community structure. The incremental clustering procedure also allows sequences with incomplete taxonomic classification to be positioned in the taxonomic tree, allowing for higher resolution in compositional comparisons of microbiome studies. Our novel tool, TIC, is offered as a complete set of scripts, allowing researchers to perform a thorough analysis from raw reads to compositional tables for subsequent comparisons (e.g., in alpha- and beta-diversity, *etc*.) within a single pipeline.

## 2 Materials and Methods

### 2.1 Overview

The TIC-Pipeline consists of a setup (i.e., installation) and four processing steps: *1*) Processing raw reads from a study’s FASTQ files, *2*) Extraction of the consensus 16S region, creation of zOTUs, and taxonomic classification up to the genus level, *3*) De-novo clustering based on taxonomic information (TIC) of the used zOTUs, *4*) Reporting the results from all the previous steps.

#### 2.1.1 Pipeline Installation

The TIC-Pipeline is a mixture of bash commands, python, and R scripts connected by a main python script. An installer script handles the installation of the command-line tools and their dependencies. The installer also downloads the reference databases (SILVA v.138), and the necessary programs, which includes KronaTools v.2.8 (Ondov et al., 2011), rapidNJ v.2.3.2 (Simonsen et al., 2010), SINA v.1.7.2 (Pruesse et al., 2012), SortMeRNA v.2.1 (Kopylova et al., 2012), USEARCH v.10 (Edgar, 2010), and VSEARCH v.2.13.4 (Rognes et al., 2016). In addition, the installer uses a dedicated file-server hosting the tools and the databases to set up dependencies for the pipeline, guaranteeing availability without breakages. Taken together, users just run the installer script, which installs R libraries and the Python packages needed automatically. After installation, we suggest a test run to ensure that all dependencies are met.

The structure of the pipeline is modular. An easy to modify text file “config options.txt” contains the configuration options controlling the pipeline’s flow. Configuration options include the number of threads to use, the current active mode (production or testing), or the input files’ location. The user may also execute each pipeline’s step independently, given that they provide correctly formatted data. For example, RDP classifications (Wang et al., 2007) could be used instead of the default SINA classifier. Detailed documentation of each option for all steps is given at the tool repository. Illustrations shown in the present manuscript directly correspond to generated outputs from the TIC-Pipeline.

#### 2.1.2 Sample-wise Processing

At this step, raw sequencing data are processed to obtain unique amplicon sequences. Those are the basis for any downstream analysis. This process includes sequence trimming to remove primers, merging paired reads, and filtering sequences based on expected error thresholds. Default options are indicative only and users are expected to fine-tune the parameters according to their needs.

#### 2.1.3 Overlapping Regions Detection and Taxonomic Classification

**FIGURE 3:**
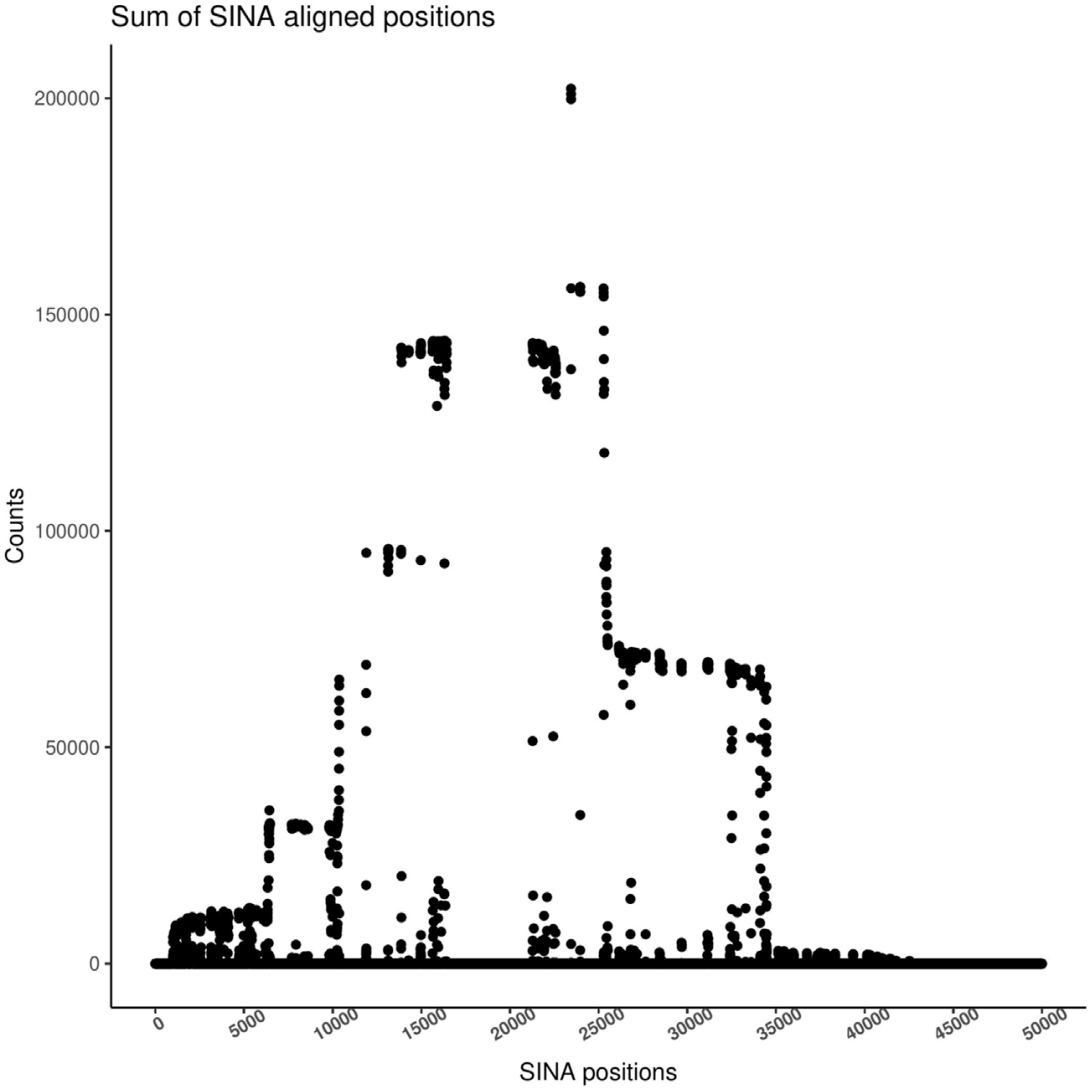
Sum of bases on each SINA alignment position. The height identifies the region with the most coverage in the coverage plot. Guided by this plot, users should select the target region for their analysis. All sequences will be trimmed around those positions, and only those containing a sufficient number of bases will be passed to the next step.

Sequences from different studies cannot always be directly compared as usage of different V-regions of the 16S rRNA genes results in sequences of different lengths and sometimes non-overlapping V-regions, which cannot be integrated. Matters are further complicated, even for sequences originating from the same method, due to the usage of diverse primers (and despite using the same V-regions) among studies. Reference Based Alignment (RBA), like SINA, has been used in the past to tackle this problem, effectively detecting any overlapping region among sequences from various studies and focusing the analysis on regions, which are represented most often (Lagkouvardos et al., 2014). Every sequence in the input dataset is aligned to the reference database (SILVA) producing a global alignment of 50,000 positions. By summing the number of aligned bases in each position of the multiple sequence alignment, the user can identify the most representative region, enabling the extraction of this region. The TIC pipeline provides an automatic calculation of this vector and plots the result, so the user can confidently identify the most informative region and set proper limits for the extraction of that region (Figure 3). The chosen region is used for taxonomic classification. The classification sub-process uses the Last Common Ancestor (LCA) shared by at least 7 of the 10 closest sequences in the database to place a sequence.

#### 2.1.4 Region Extraction and Denoising

Based on the user’s evaluation of the region with the highest coverage among samples (recorded as parameter in the configuration file), each sequence is trimmed for positions outside this defined region. Trimmed sequences are pooled, dereplicated, and denoised using the UNOISE3 algorithm (Edgar, 2016) to create zOTUs. All denoised sequences are checked for valid 16S rRNA sequences by SortMeRNA using the SILVA bacteria and archaea databases. The taxonomic information derived from the previous step is added automatically to the header of the zOTU FASTA file and is used in the next step to guide the clustering.

#### 2.1.5 Taxonomy Informed Clustering

**FIGURE 4:**
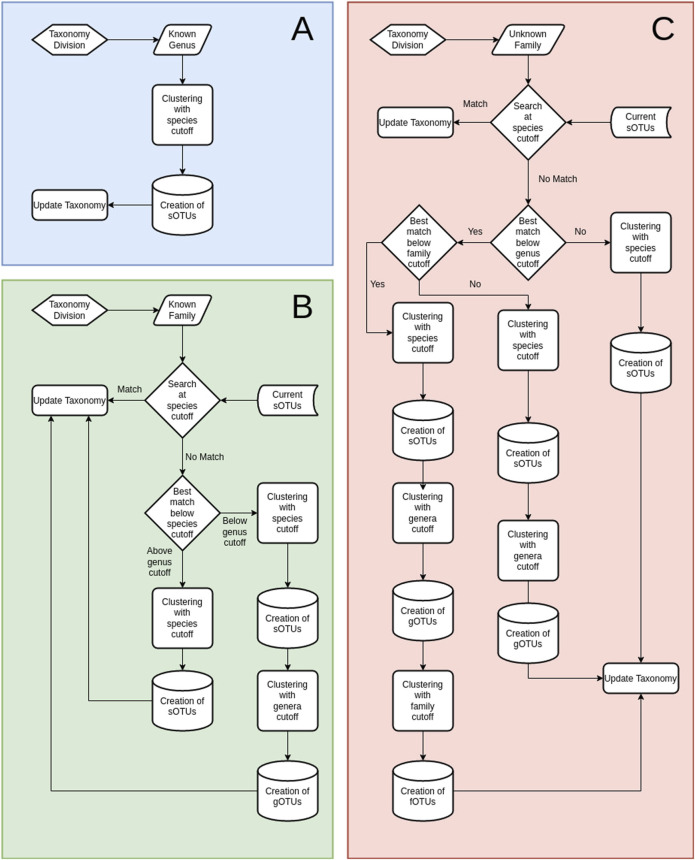
Overview of the TIC processes. **(A)** Diagram of the TIC process for sequences with identified genus-level taxonomy. All sequences within each genus are used to create sOTUs. **(B)** Diagram of the TIC process for sequences with identified taxonomy up to the family. First step is searching for matches among those sequences and sOTUs contained within genera in the current family. Not matched sequences create novel sOTUs, which are searched for matches at the genera cutoff level (default 95%), as specified at the configuration file; if not matched again, they produce novel gOTUs. Any matched sequence gets the taxonomy of its match. **(C)** Diagram of the TIC process for sequences without family classification. Searching for matches among existing sOTUs at the order level. Not matched sequences create novel sOTUs, which are searched for matches at the genera cutoff level; if not matched again, they produce novel gOTUs. Another search is conducted afterwards at the user-specified family similarity percentage (default 90%), afterward, and if not matched again, novel fOTUs are created. Any matched sequence gets the taxonomy of its match.

A step-wise taxonomy-guided clustering was implemented to utilize position-specific taxonomic information for purer clusters. TIC’s starting point is the pool of unique denoised sequences (zOTUs) with a recognized genus name (Gseqs). Gseqs are clustered within each genus to produce molecular species (sOTUs) within the identified genera. Afterwards, sequences that have been classified only up to the family level (Fseqs) are processed. In order to account for limitations in the taxonomic classification (i.e., missing levels), such Fseqs are first searched if they match any existing sOTU from Gseqs within the current family. In case a match is found, the taxonomy of the zOTU in question is updated if within a designated species cutoff level. However, sequences matching existing sOTUs above the designated genus cutoff level, but below the species level, are assumed to be novel sOTUs within the existing genera. Finally, Fseqs without a match, even at the genus level to existing sOTUs, are used to produce novel sOTUs that are next clustered again to novel gOTUs. Sequences with an unidentified taxonomic family follow the same procedure as before, but with the added layer of fOTUs. For instance, they are first matched against sOTUs, gOTUs, and known families of the same order. If no matches are found according to corresponding cutoffs, the unidentified sequences are designated as novel fOTUs (Figure 4).

Since there is no consensus on sequence similarity values between orders, classes, and phyla across all bacteria, TIC produces only novel fOTUs, gOTUs and sOTUs, while filling the other missing taxonomic ranks (i.e., phylum, class and order) with a placeholder, i.e., UNKPHYLUM, UNKCLASS, and UNKORDER, respectively. Since no universal cutoffs for 16S rRNA gene fragments (i.e., amplicons) exists for delineating species, genera, and families, we adopt the popular cutoffs normally used for the whole 16S rRNA molecule (97, 95, and 90%, respectively). However, we recommend that those cutoffs be tailored to each analysis to reflect the variance captured within the selected fragment (i.e., V-region used).

#### 2.1.6 Results Reporting and Graphs

**FIGURE 5:**
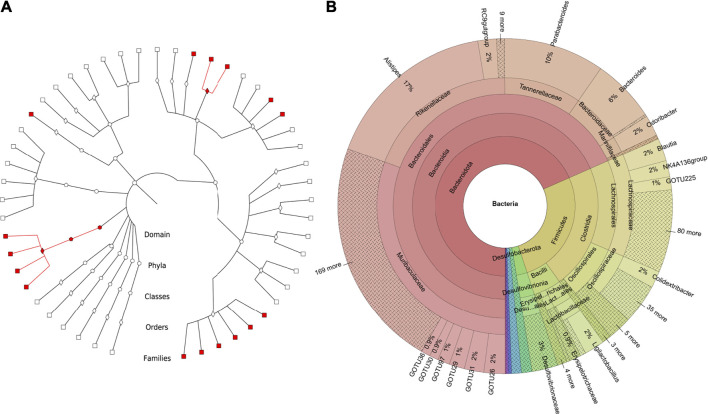
Plots produced from the TIC-Pipeline from the mouse dataset of Muller et al. **(A)** Graphlan plot depicting the taxonomic tree of the denoised sequences after TIC incorporated both novel (red) and known (white) clades up to the family level. **(A)** Krona plot quantifies the size of each taxonomy in the merged study samples. Contains novel and known taxonomies as produced by the SINA classifier and TIC.

The produced zOTUs are outputted in FASTA format with the full taxonomic path up to sOTU level, incorporating any novel families and genera in the header of each zOTU. Furthermore, the taxonomic tree (Figure 5A) indicates novelty (i.e., unknown bacteria and archaea) within the given study by color-coding each branch (Asnicar et al., 2015). Towards this end, the microbial novelty and diversity in the examined samples are displayed by uniting the taxonomic tree and the quantification information contained within the Krona plot in a combined figure (Figure 5B). Finally, the zOTU table produced shows how many reads in each sample constitute the respective sOTU together with the sOTUs’ taxonomy. Additional mapping files produced as output reflect the relations between sOTUs to gOTUs and gOTUs to fOTUs.

### 2.2 Benchmarking

#### 2.2.1 Naive Classifiers *vs* TIC

Comparisons between naive classifiers (USEARCH and VSEARCH) and TIC require a dataset for which the complete taxonomic information is available. We created a dataset fulfilling this requirement by using the sequences from the Living Tree Project (Yarza et al., 2008) and their corresponding similarity matches at a threshold of 98% of the non-redundant SILVA v128 database. This dataset was designated LTP. All sequences included were classified with SINA and, in order to simulate real-life scenarios, we pruned the produced taxonomies with two strategies, designated “hard” and “soft.” Hard pruning corresponds to the removal of whole clades from the taxonomic tree at random levels. We removed about 10, 5, 2.5 and 1% from the tree at the level of genera, family, order, and class, respectively. This hard pruned dataset was used to test the performance for cases where completely unknown taxonomic groups are present within the actual data. The chosen percentages were based on empirical observations on missing taxonomic classification at each level when using SILVA on real data. The second pruning strategy “soft” is the stochastic removal of taxonomic information, simulating shortcomings of the classification process in assigning taxonomies to every leaf of each clade correctly, also following the above percentages. Those strategies are needed because LTP consists only of taxonomically known sequences on which classifiers have an advantage. Our pruning strategies allow a fair comparison between the taxonomy-aware TIC and the naive clustering algorithms. For testing, the clustering was performed 100 times for each strategy and tool.

#### 2.2.2 Clustering Metrics

The following metrics were calculated for every trial: cluster purity, Adjusted Rand Index (ARI), and Normalized Mutual Information (NMI). Concerning cluster purity, this value ranges from 0 to 1. It shows the mean fraction of sequences across all clusters that are correctly pooled together according to the genus taxonomic information included in LTP. Next, the ARI gives a value about how often a randomly chosen sequence from the dataset was found in the same cluster as in the original LTP data set, when producing the same clustering (Steinley, 2004). Finally, the Normalized Mutual Information (NMI) quantifies the amount of information we obtain from clustering A by observing the clustering B; thus, it is a measure on how similar two different clustering runs (i.e., A and B) are (Vinh et al., 2010). A higher NMI score indicates that the information we got by clustering reflects the original taxonomic assignments closer. In turn, this allows us to approximate the entropy of the produced clustering.

We compared execution time for TIC with USEARCH and VSEARCH, allocating eight threads on the same machine and with Debian Linux as the host operating system. Each tool was evaluated further based on the number of produced families, genera, and species. This evaluation allowed us to determine the inflation for each type of clustering in each’s diversity measures.

#### 2.2.3 Template Data

*Bifidobacteriaceae* are a group of bacteria, which are responsible for oligosaccharide metabolism in mammals. They are one of the dominant families present in the human gastrointestinal tract during infancy (Pham et al., 2016). There is a growing interest in their role as probiotics. Therefore, illuminating the microbial diversity within this family will help us evaluate the range within which we operate and potential sources of hidden diversity. The template data include 227,418 *Bifidobacteriaceae* sequences, which were classified as such by RDP classifier. These sequences have been originally detected across 11,074 samples of diverse environmental origin within IMNGS. IMNGS is a database containing currently more than 500 k samples analyzed by 16S rRNA gene amplicon sequencing. All data are preprocessed and IMNGS offers, next to other means, automated export of all sequences belonging to a selected taxonomy at once (Lagkouvardos et al., 2016). In addition to the above, to illustrate the use of TIC in microbial profiling studies based on amplicon data, we processed the dataset from Müller et al. (2016). In this study, the role of nutrition and hygiene concerning mice’s gut microbiomes was investigated. The original results demonstrated that diet and the hygiene level of the mouse facility affect the mice’s gut microbial profiles. The raw sequencing data of the study are available in ENA under accession PRJEB13041.

## 3 Results

### 3.1 Benchmarking Results

#### 3.1.1 Number of Created Taxa

**FIGURE 6:**
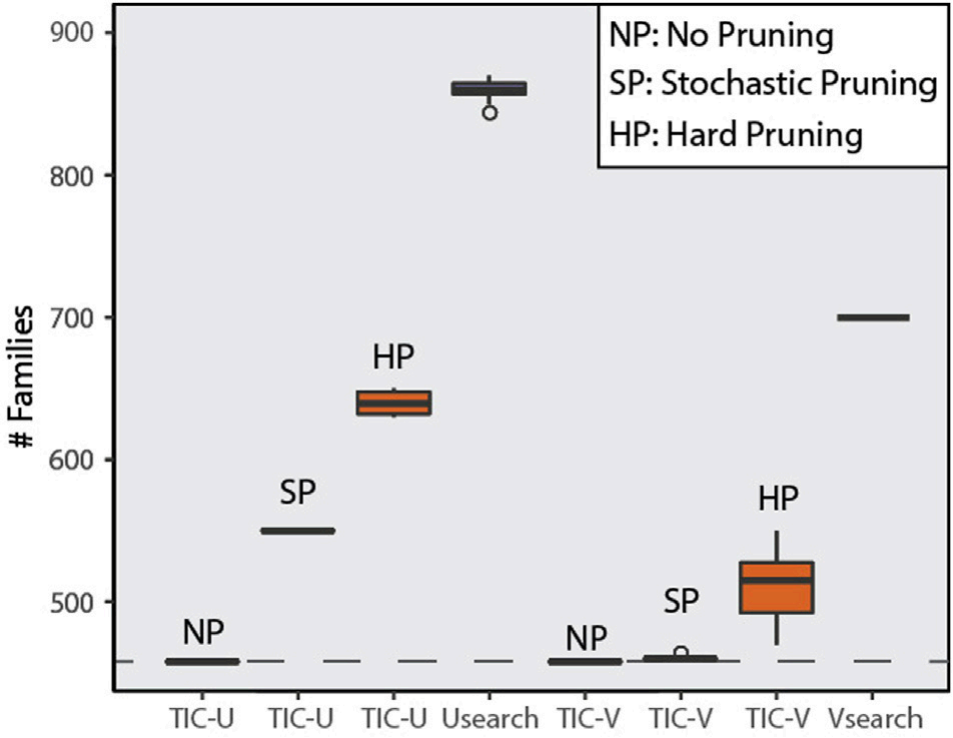
Comparison of the three tools in regards to predicted family number on the LTP dataset under different configurations. TIC was executed containing USEARCH (TIC-U) and VSEARCH (TIC-V) as the integrated clustering tool, with different modes of taxonomic pruning of the input sequences. These tools were also executed as standalone. The dashed line represents the actual number of families in the dataset. USEARCH performs worse in terms of inflation of predicted family level clusters, with VSEARCH resulting in only moderate inflation. TIC reflects this trend in its operation depending on the tool utilized, especially with hard pruning (compete for removal of assignments for whole taxonomic branches). For soft (stochastic) pruning (only removing taxonomic information for random sequences), the TIC performs significantly better than the naïve usage of the corresponding clustering tool. In cases where the classifier can successfully assign family level taxonomy to all sequences, as for the sequences in the LTP dataset, the TIC mirrors this information resulting in a perfect grouping of the sequences as expected.

The LTP dataset used contains sequences with known taxonomic assignment up to the species level, with 458 families, 1,590 genera, and 13,903 species. The TIC pipeline identified 508, 460, and 458 clusters at family level when using hard- and soft-pruned, and the complete taxonomy, respectively. In contrast, both USEARCH and VSEARCH resulted in estimations almost twice the size of the actual family numbers (Figure 6). Of note, when the complete SINA classification is available (no pruning), TIC, as expected, successfully mirrors the underlying family structure. Thus, overall, values produced by TIC are the closest reflection of the ground truth we could get.

The results for the genus level reconstruction showed that, when taxonomic information is missing (e.g., due to novel taxa or incomplete classifications), TIC and VSEARCH perform similarly. In contrast, USEARCH inflates genus numbers (Supplementary Figure S1). Since TIC is the only tool utilizing taxonomy knowledge, the results match the initial genus composition as expected in the no pruning scenario. All three tools fail to recapture the species diversity contained within the dataset (Supplementary Figure S2). We suggest that this is because taxonomic species definitions are not solely based on 16S rRNA gene sequence similarity, and none of the tools can account for this external information. However, TIC calling USEARCH produces species cluster closer to the ground truth regardless of the pruning scenario, while the TIC with VSEARCH improves its performance significantly when compared with default running of VSEARCH.

#### 3.1.2 Quality of Created Taxa

Clusters produced by TIC are purer than those produced by USEARCH and VSEARCH (Tables 1, 2). Since TIC uses taxonomic information, unwanted merging of sequences originating from distant taxonomies is less likely, while other tools are blind to taxonomy and, thus, combine unrelated sequences solely based on similarity thresholds. Although VSEARCH produces a higher ARI score, this stems from the inflation of the number of produced species, genera, and families in combination with the rigorous approach taken when calculating pairwise similarity scores, resulting in many one-member clusters that should have been merged otherwise. The NMI score calculated for all tools is almost identical. Therefore, this value should not be viewed in isolation. Taken together, across all metrics tested here, TIC is the better choice.

**TABLE 1:** Clustering quality comparison among tools.

Level	Scenario	Purity	ARI	NMI
Species	TIC_Stohastic_VSEARCH	**0.99**	0.93	0.97
	TIC_Stohastic_USEARCH	**0.99**	0.93	0.97
	TIC_Hard_VSEARCH	**0.99**	0.93	**0.98**
	TIC_Hard_USEARCH	**0.99**	0.93	0.97
	USEARCH	0.98	0.88	0.97
	VSEARCH	0.97	**0.97**	0.97
Genera	TIC_Stohastic_VSEARCH	**1**	0.93	0.97
	TIC_Stohastic_USEARCH	**1**	0.93	**0.98**
	TIC_Hard_VSEARCH	**1**	0.93	**0.98**
	TIC_Hard_USEARCH	**1**	0.93	0.97
	USEARCH	0.87	0.88	0.97
	VSEARCH	0.93	**0.97**	0.97
Families	TIC_Stohastic_VSEARCH	**1**	0.93	0.97
	TIC_Stohastic_USEARCH	**1**	0.93	0.97
	TIC_Hard_VSEARCH	**1**	0.93	**0.98**
	TIC_Hard_USEARCH	**1**	0.93	0.97
	USEARCH	0.97	0.88	0.97
	VSEARCH	0.88	**0.97**	0.97

Regardless of the pruning method, taxonomic level, and the underlying tool, the TIC creates better clusters in terms of purity and the NMI statistic. VSEARCH inflates the number of clusters and, in conjunction with its no-heuristic approach when calculating the sequence pairwise identity score, results in higher ARI scores. Although USEARCH uses heuristics for this calculation, the TIC restrains it, thus keeping the ARI score high. Maximum values are highlighted (bold) for each column for each level.

**TABLE 2:** Level of impurity for genus and family level clusters created by USEARCH and VSEARCH compared with the TIC approach for the LTP dataset.

Tool	Species	Mixed genera (percentage)	Mixed families (percentage)
USEARCH	6,668	299 (08.20)	115 (11.50)
VSEARCH	5,817	179 (05.84)	83 (08.80)
TIC_Soft_VSEARCH	5,824	0	0
TIC_Soft_USEARCH	6,315	0	0
TIC_Hard_VSEARCH	5,839	0	0
TIC_Hard_USEARCH	6,371	0	0

Impurity was calculated as the number of genera/families containing LTP sequences with conflicting taxonomic backgrounds. Both naive clustering tools result in more than 5% of genera and 8% of families having impure composition.

#### Performance

**FIGURE 7:**
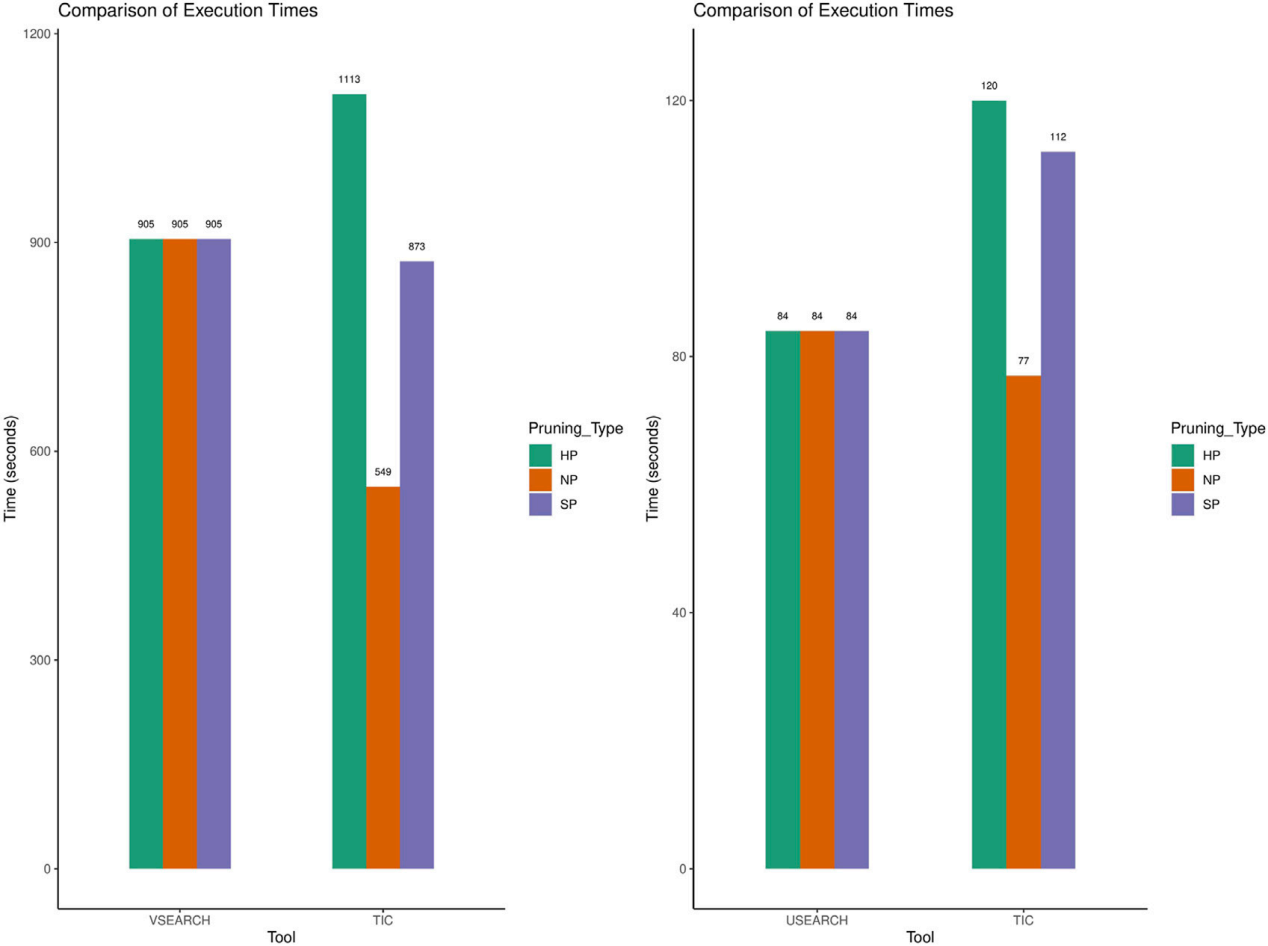
Comparison of execution times for VSEARCH, USEARCH, and TIC running with each as an underlying tool respectively. Although slower, TIC is comparable with either tool regardless of the pruning method.

The computational speed for TIC is primarily dependent on the underlying tool. TIC manages to offset the required time to handle taxonomic information by clustering smaller subsets of data created from the taxonomy classification (Figure 7). Performance is further affected by the rate of available taxonomic information and no-pruning run times are always shorter than those from simulations, including partial classifications.

### 3.2 Template Results

#### 3.2.1 Amplicon Showcase

The Müller dataset (Müller et al., 2016) contains 238,936 raw sequences produced from 24 samples. This dataset contains 6,580 unique sequences after extraction of the representative region (i.e., SINA alignment positions: 6,500–22,500, number of bases: 384), trimming around it, and denoising to zOTUs. Taxonomic classification using the integrated SINA classifier with SILVA as the reference database resulted in 319 and 2,412 unclassified zOTUs for family and genus level, respectively. Clustering those sequences to form molecular species (sOTUs) using TIC or the two naive clustering tools (i.e., USEARCH, VSEARCH) resulted in similar sOTU numbers ( ≈1380 ; Table 3). However, 78 and 83 out of the predicted sOTUs created by USEARCH and VSEARCH, respectively, contain zOTUs with non-matching taxonomic assignment, strongly suggesting impure clusters. Moreover, 656 and 694 sOTUs created by USEARCH and VSEARCH respectively, have incomplete taxonomic assignments when we follow the old paradigm of clustering first and assign taxonomy later. For instance, 153 and 150 sOTUs produced by USEARCH and VSEARCH, respectively, have not been assigned to any family, while 503 and 544 sOTUs, respectively, have family classification only, but were not assigned to a genus (Table 3).

**TABLE 3:** Comparison of TIC with naïve clustering approaches on microbial amplicon data from mice.

Tool	Predicted SOTUs	No genus assigned	No family assigned
USEARCH	1,378	656	153
VSEARCH	1,380	694	150
TIC-Pipeline	1,279	—	—

Nearly 700 sOTUs produced by naïve *de novo* clustering have the missing genus-level classification, and around 150 of those could not be assigned to a known family. The TIC organizes those sequences to 356 novel gOTUs and introduces 16 novel fOTUs.

In contrast, TIC organized unclassified sOTUs in many cases within distinct gOTUs of a given family. Such unclassified sOTUs would otherwise be collectively treated as unknowns or even discarded. Similarly, for four taxonomic orders containing sOTUs with unknown family assignments, TIC stratified the sequences in appropriate fOTUs, further enhancing the insights into the community’s structure of this dataset.

#### 3.2.2 Diversity Showcase

**FIGURE 8:**
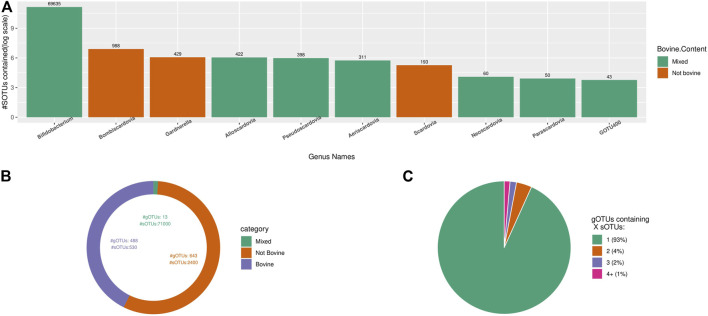
Overview of the environmental origins of the *Bifidobacteriaceae* sequences grouped in gOTUs. **(A)** Rank order of 10 most diverse gOTUs, differentiated by the origins of their constituent sOTUs. *Bifidobacterium* is by far the most diverse genus of this family. **(B)** High niche specificity of *Bifidobacteriaceae* gOTUs contained within the bovine samples. **(C)** Pie chart indicating the size of gOTUs created by TIC from all available sequences classified as belonging to the *Bifidobacteriaceae* family extracted from IMNGS.

For testing about diversity outcomes when applying TIC, the used dataset contains only sequences within the *Bifidobacteriaceae* family as identified by the RDP classifier (v.2.11 with training dataset 16) included in the IMNGS database. We re-classified the retrieved sequences using SINA and the latest online RDP classifiers (training dataset 18), removing all sequences not classified as *Bifidobacteriaceae*. After identifying the most representative region in this dataset (i.e., SINA alignment positions: 12,000–25,300, number of bases: 288), trimming and dereplication, almost 75,000 unique denoised sequences remained. The produced dataset was processed with TIC, resulting in about 72,000 molecular species organized in about 1,100 gOTUs. The known genus *Bifidobacterium* has about 69,000 sOTUs, reflecting the total molecular species diversity. The rest of the nine described genera from the *Bifidobacteriaceae* have 2,876 sOTUs, with an average of 320 sOTUs per genus (Figure 8A). The 1,134 remaining novel gOTUs contain only a single sOTU (Figure 8C). Comparing TIC to the other naive clustering algorithms shows again an inflation of the numbers of species and genera cluster formed (Table 4). Furthermore, both similarity-based tools separated the *Bifidobacteriaceae* sequences into 1000s of new families, while TIC kept them as one family.

**TABLE 4:** Diversity estimations among the three tools for the *Bifidobacteriaceae* sequences extracted from IMNGS.

Tool	Species number (k)	Genera number (k)	Families number
USEARCH	62	35	2.8 k
VSEARCH	52	28	3.5 k
TIC-Pipeline	52	1.1	1

Denoised sequences were clustered with the three tools. Using VSEARCH for within branch clustering, The TIC produces the most conservative results and should be used as a baseline.

About half of the discovered gOTUS (1.1 k) incorporate sequences originating solely from bovine samples, with only 13 gOTUs (which include most of the already described genera) containing sequences from diverse origins. The other half of the gOTUs consist exclusively of sequences of non-bovine origin (Figure 8B), including the genera *Bombiscardovia*, *Scardovia*, and *Gardnerella* that were not found in any of the bovine samples used in our analysis.

## 4 Discussion

### 4.1 Amplicon Studies Integration Is Problematic due to Partially Overlapping Targeted Regions

Selection of different hypervariable regions for each amplicon-based experiment inevitably results in different primer sets used in different studies (Schloss et al., 2011), (Liu et al., 2008). The absence of a consensus (Abellan-Schneyder et al., 2021) of the scientific community on which region should be targeted for a given purpose further complicates this issue (Li et al., 2014), (Dassi et al., 2014). Such diverse primer designs prohibit the effortless integration of amplicon studies even in the absence of other experimental differences. In such cases, the suggested procedure is to identify a common region across studies, when such a region exists, and trim all sequences accordingly (Lagkouvardos et al., 2016). The proposed TIC pipeline follows this idea by using the SINA aligner. Extracting the region of overlap for different studies and collapsing gaps (which are inserted otherwise for better alignments) makes the sequences compatible and allows us to analyze samples processed with different, but overlapping V-regions together. Currently, the selection of the common region is performed by manual inspection, but an automated procedure is in development.

### 4.2 Naive Classification Tends to Produce Impure Clusters

Naive clustering tools are based solely on sequence similarity in creating groups. In contrast, TIC enhances the clustering process by utilizing the taxonomic information of each sequence acquired beforehand. The metrics tested here, clustering purity, ARI, and NMI, show that TIC outperforms both USEARCH and VSEACH (Table 4).

Fixed similarity levels cutoffs used for clustering will not always produce clusters that correspond to valid taxonomic paths (Edgar, 2018), (Schloss and Westcott, 2011), (White et al., 2010), (Huse et al., 2010). New approaches to clustering have been proposed, based on machine learning and other methods, but they have not yet seen widespread adaptations (James et al., 2018) (Eren et al., 2015), (Navlakha et al., 2010), (Preheim et al., 2013), (Mahé et al., 2014). The accuracy of similarity-based tools can be improved by introducing clade specific similarity cutoffs. For such an approach, phylogenetic distances of all described taxa could be used to generate clade-specific similarity limits reflecting the average distance of taxonomic units (e.g., average distances among sequences from all genera within a family to set the genus similarity cutoff for that family). These limits should be further refined based on the selected region of the 16S rRNA gene used in each study.

In any case, all tools tested struggle mapping sequences to the underlining taxonomic delineation for species-level clusters. The main reason is that taxonomic nomenclature, especially at the species level, is not necessarily reflected in adequate differences in the 16S rRNA gene. Instead, functional characteristics, phenotypes, or pathogenicity differences of the bacteria are used to designate species. A well-known example is the *Escherichia-Shigella* clade, with otherwise almost identical 16S rRNA genes, but even different genus names. Other such examples exist. Thus, since classifiers based on 16S rRNA cannot (yet) assign taxonomy up to the species level, TIC cannot overcome the absence of this information in the molecular species-level prediction. In any case, all classification tools finally rely on reference databases that affect their performance. That is why the usage of the latest and most comprehensive iteration is the recommended practice.

Naive similarity-based clustering tools’ results are affected only by their underlying algorithm regardless of other available information. In contrast, TIC’s performance is bound to the completeness of the classifier-provided taxonomic information. Already with the current level of knowledge extractable from commonly used classifiers, TIC outperforms naive clustering tools despite some novel sequences existing in most studies. Furthermore, as the classifiers improve in their capacity to translate sequence signatures to finer taxonomic classifications, TIC-produced clusters will also be affected and improved in terms of purity and quality.

### 4.3 Evaluation of Taxonomy Informed Clustering in Single Amplicon Studies

There has been a growing tendency to abandon “traditional” OTUs based pipelines due to their problems in clusters purity, reproducibility, and interoperability in favor of denoised sequences. Denoised sequences are called with different names depending on the tool used (e.g., ASVs, zOTUs). Although there are clear benefits in such pipelines using denoised sequences, limiting processing to the molecular strain level also is often problematic. To the extreme, a strain can be any single bacterium differing by a single mutation across its genome, effectively accounting for nearly as many strains as individual bacteria in a sample. However, commonly “strains” are viewed as relatives belonging to a given species and differing in few to several phenotypic characteristics. In any case, strains are not well defined, especially when derived by molecular sequences. Sequence fragments of the 16S rRNA gene of, e.g., about 300 bases may be identical and, therefore, different strains are assigned to one amplicon variant, although originating from several. Increasing the length of the fragment, e.g., by different sequencing technologies, may reveal an increased number of strains/variants for the same sample. Therefore, alpha-diversity measures based on denoised sequences of different lengths offer a non-comparable sample diversity measure only. Common OTU clustering of 16S rRNA genes to a fixed similarity cutoff for accommodating molecular species is more defined, stable across sequencing lengths and technologies and, reflects a more meaningful ecological entity. However, other problems with OTUs, as mentioned above, exist. Concerning, beta-diversity measures similar problems arise. For instance, methods like Jaccard and Bray-Curtis do not consider the similarity (i.e., taxonomy) among the different strains in a sample and, therefore, tend to inflate the distances across microbial profiles between samples. Finally, it defeats its purpose when studies perform strain-level processing at first, but use the binned family abundances or even higher taxonomies for their comparisons. In contrast, TIC offers an incremental, structured dissection of the sequencing outputs from zOTUs to sOTUs, and then proceeds to gOTUs and fOTUs. Since the taxonomic placement of the sequence is clear, exploring the different hierarchical levels is easy depending on the question. For instance, the test run using real amplicon data showed that multiple well differentiated fOTUs and gOTUs were revealed. These would otherwise be collectively treated as unknowns and not contribute to understanding a sample’s or experiment’s ecology. Clearly, the refined taxonomic classification of every sequence assists downstream comparisons among higher taxonomic levels and reveal differential patterns across yet undescribed groups. Since taxonomy-informed clustering always results in purer clusters and more informative outcomes, we strongly recommend integrating tools like TIC in future amplicon analysis pipelines.

### 4.4 Diversity Analysis of *Bifidobacteriaceae*

The *Bifidobacteriaceae* family has attracted much interest due to its mostly positive effects on humans and other mammals. Microbes of this family colonize the infant gut, aiding in nutrient absorption (Turroni et al., 2011) and can act as probiotics with beneficial effects in patients with irritable bowel syndrome (Yuan et al., 2017) and other intestinal diseases (Matsuoka et al., 2018). This family is currently composed of 10 genera containing 124 valid species in taxonomic nomenclature. Specifically, the genus *Bifidobacterium* covers most of the family’s diversity with 105 species, representing the most diverse genus of the *Bifidobacteriaceae*. This genus is most frequently associated with the gastrointestinal tract of humans (Scardovi and Trovatelli, 1974). However, molecular evidence has shown the presence of *Bifidobacterium* in other niches beyond the mammalian gut (Watanabe et al., 2009), (Dong et al., 2000). Species within the *Bifidobacteriaceae* show varying degrees of ecological adaptation with few cosmopolitan taxa within an otherwise specialized majority. This is due to the intense selective pressure for acquiring and retaining genes responsible for utilizing various carbohydrates to compete in their respective ecological niches (Milani et al., 2014), (Milani et al., 2015).

Our findings indicate an even larger *Bifidobacterium* genus, followed by also prolific, but less known genera and numerous novel candidate genera. Interestingly, the distribution of the novel genera and species detected here by molecular data seems to follow the distribution of currently known and described species within the recognized genera (normalized chi-square *p*-value: 0.12). It is safe to assume that part of this discrepancy in species numbers (i.e., known vs unknown) is attributed to uneven sampling and isolation efforts devoted to human and mammalian gut environments in general, which the genus *Bifidobacterium* seems to dominate. Nevertheless, the observed pattern is so pronounced that it calls for further research to unravel the ecological constraints that dictate this massive differentiation of *Bifidobacterium* and the modes of persistence and dispersal of this vital family of bacteria in contrast to the other genera in this family.

## 5 Conclusion and Future Work

The TIC pipeline is a modular set of tools that facilitate fast and easy analysis of microbial data to produce the data files most commonly used in microbial ecology. In the present manuscript, we demonstrate the advantages of reversing the current practice of *de novo* sequence clustering followed by taxonomic classification. In contrast, taxonomically placed sequences allow utilizing the classifier’s information in guided clustering and this approach results in higher cluster quality and purity, and allows proper placing of yet unassigned sequences in the taxonomy.

Currently, the TIC pipeline will soon be integrated in online analytical services while further simplifying the technical requirements for users. New features and outputs, such as making the TIC pipeline available to distributed systems, enhanced graphical representations, and other features, which can be requested by the community, will be added.

## Data Availability

The datasets presented in this study can be found in online repositories. The names of the repository/repositories and accession number(s) can be found in the article/Supplementary Material.
