# Supplementary material for: Taxonomy Informed Clustering, an Optimized Method for Purer and More Informative Clusters in Diversity Analysis and Microbiome Profiling
Source: Front Bioinform. 2022 Apr 27;2:864597. doi: 10.3389/fbinf.2022.864597 (PMC9580952; doi:10.3389/fbinf.2022.864597)
Supplement: Supplementary file 1 [file DataSheet1.pdf]

Supplementary Material

1 SUPPLEMENTARY TABLES AND FIGURES

1.1 Figures

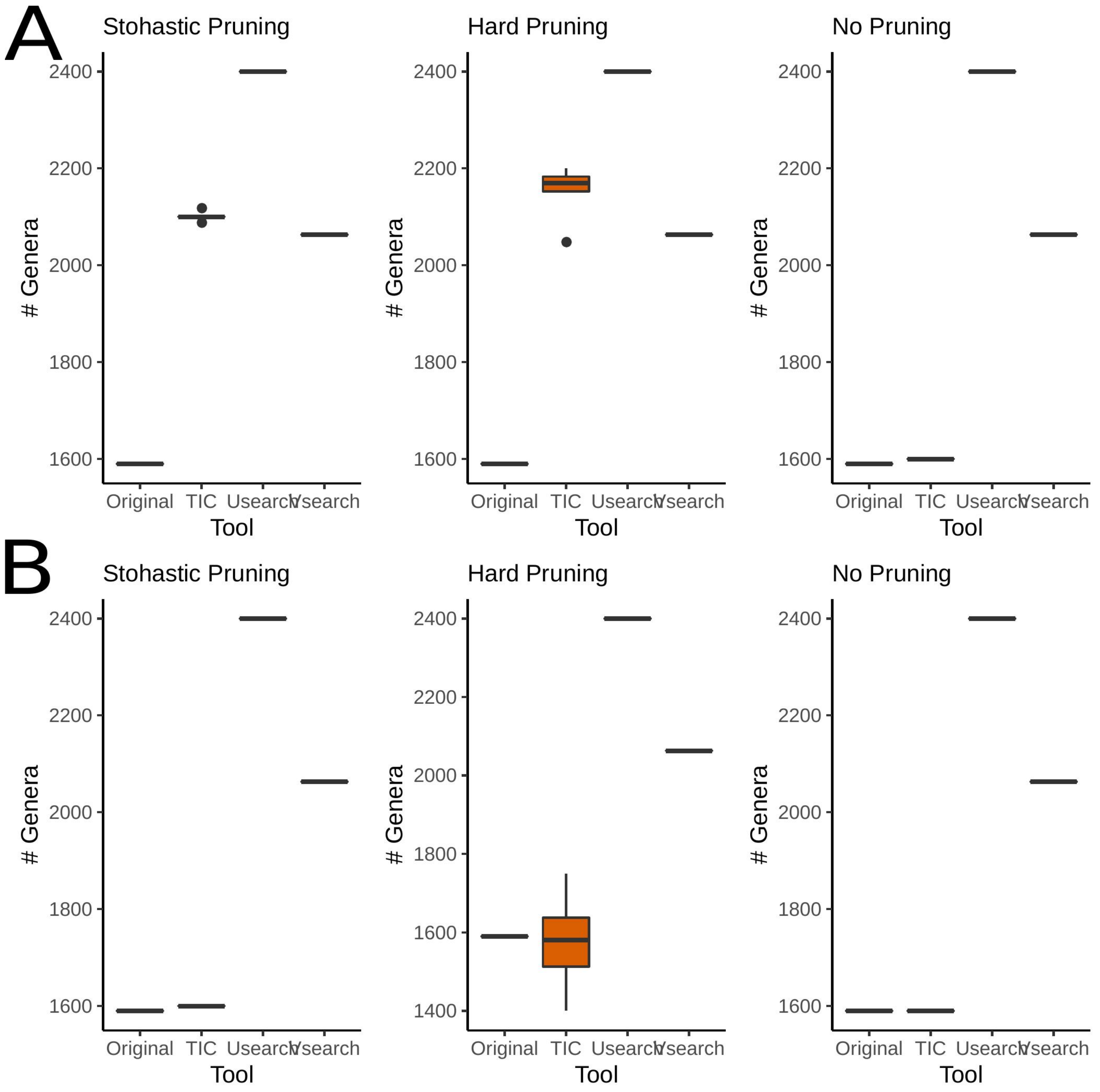

Figure S1. Comparison of the three tools in regards to produced genera numbers. USEARCH performs worse in terms of inflation of predicted genus level clusters with VSEARCH resulting in only moderate inflation. TIC reflects this trend in its operation when each tool is called. **(A)** TIC output while using USEARCH as the underlying tool. **(B)** TIC outputs with VSEARCH as the underlying tool.

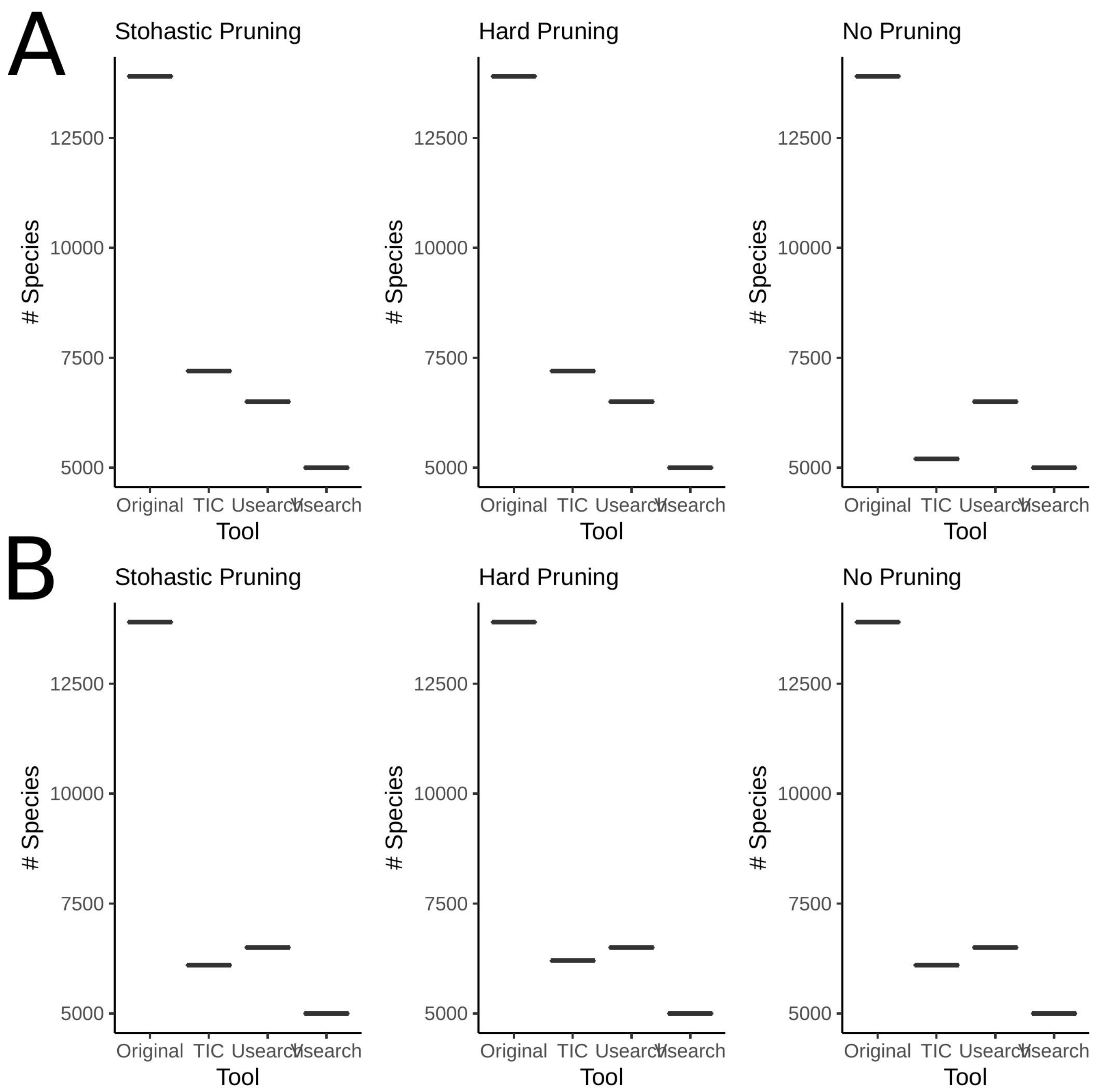

Figure S2. Comparison of the three tools in regards to produced species numbers. Similarity-based clustering failed to reproduce the information of the taxonomic species existing in the dataset. **(A)** For USEARCH-based TIC additional taxonomic constrains resulted in lower estimated number of species. **(B)** For VSEARCH-based TIC, pruning scenario had no effect in the estimated number of species.

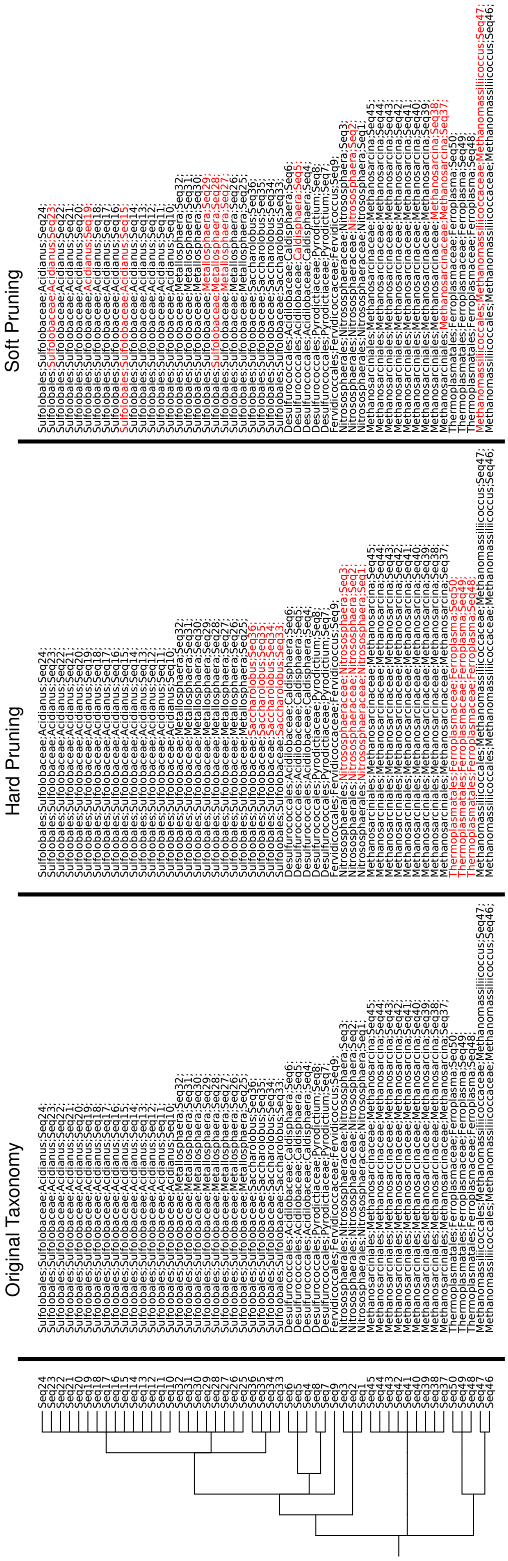

Figure S3. In hard pruning whole taxonomic clades are removed. In this example, the genus *Saccharolobus*, the family *Nitrososphaeraceae* and the order *Thermoplasmatales* were removed. Whereas in soft pruning, each taxonomy is pruned by chance for the different levels as described in the main text.

```
>M00202:72:000000000-A85MG:1:1102:22476:17298;tax=Bacteria;UNKPHYLUM;UNKCLASS;UNKORDER;FOTU16;GOTU328;SOTU1246;
TGGTCAATGGGCGCGAGCCTGAACCAGCCAAGTCGCGTGAGGGAGGACGGCCCTACGGGTTGTAAACCTCTTTTGCCGGGGAGCAACGGGCGTCACGTGTGGCGCCACTGA
GAGTACCCGGAGAAAAAGCATCGGCTAACTCCGTGCCAGCAGCCGCGGTAATACGGAGGATGCGAGCGTTATCCGGATTTATTGGGCGTAAAGAGTACGTAGGCGGTTCTT
TAAGCGCAGGGTTTAAGGCGATAGCTTAACTATCGTTCGCCCTGTGAACTGGGGGACTTGAGTATCGGAGAGGAAAGCGGAATTCCTAGTGTAGCGGTGAAATGCGTAGAT
ATTAGGAGGAACACCAGTGGCGAAGGCGGCTTTCTGGACGAAAACTGACGCTGAGGTACGAAAGCGTGG

>M00202:72:000000000-A85MG:1:1103:20277:14211;tax=Bacteria;UNKPHYLUM;UNKCLASS;UNKORDER;FOTU14;GOTU310;SOTU1219;
TGGTCAATGGGCGGGAGCCTGAACCAGCCAAGTCGCGTGAGGGAAGACGGTCCTATGGATTGTAAACCTCTTTAGGCGGGGAGCAATGCCGGGCACGCGTGCCCGGAGGGA
GAGTACCCGCAGAATAAGCATCGGCTAACTCCGTGCCAGCAGCCGCGGTAATACGGAGGATGCGAGCGTTATCCGGATTTATTGGGCGTAAAGCGTTCGTAGGCGGCAAGG
TAAGTCTGATGTTAAAGCCCGGGGCTCAACTCCGGTTCGGCATTGGATACTATCTAGCTAGAATGTGGTAGAGGTAAAGGGAATTCCTGGTGTAGCGGTGAAATGCGTAGA
TATCAGGAGGAACATCGGTGGCGAAAGCGCTTTACTGGACCATTATTGACGCTGAGGAACGAAAGCCAGG

>M00202:72:000000000-A85MG:1:1106:5290:12199;tax=Bacteria;UNKPHYLUM;UNKCLASS;UNKORDER;FOTU13;GOTU308;SOTU1215;
TCCACAATGGGCGAAAGCCTGATGGAGCAACGCCGCGTGGGTGAAGAAGGTCTTCGGATCGTAAAACCCTGTTGTTAGAGAAGAAAGTGCGTGAGAGTAACTGTTCACGTT
TCGACGGTATCTAACCAGAAAGCCACGGCTAACTACGTGCCAGCAGCCGCGGTAATACGTAGGTGGCAAGCGTTATCCGGATTTATTGGGTTTAAAGGGTGCGTAGGCGGA
TTGATAAGTTAGAGGTGAAATGTCCGAGCTCAACTCGGGAACTGCCTCTAATACTGTTGATCTAGAGAGTAGATGCGGTAGGCGGAATGTATGGTGTAGCGGTGAAATGCT
TAGAGATCATACAGAACACCGATTGCGAAGGCAGCTTACCAATCTATATCTGACGTTGAGGCACGAAAGCGTGG

>M00202:72:000000000-A85MG:1:1101:10399:15447;tax=Bacteria;Firmicutes;Clostridia;UNKORDER;FOTU11;GOTU292;SOTU1186;
TCCGCAATGGGCGCAAGCCTGACGGAGCAATGCCGCGTGAACGAAGAAGGTCTTCGGATTGTAAAGTTCTGTCCTTATCGAAGAGAGGGTATAGAGTGAAAAATGATATAC
TAGGACGGTAGATGAGGAGGAAGCTCCGGCTAACTACGTGCCAGCAGCCGCGGTAATACGTAGGGAGCAAGCGTTGTCCGGATTTACTGGGTGTAAAGGGTGCGTAGGCGG
CCTTGCAAGTCAGAAGTGAAATCCATGGGCTTAACCCGTGAACTGCTTTTGAAACTGTAGGGCTTGAGTGAAGTAGAGGCAGGCGGAATTCCCGGTGTAGCGGTGAAATGC
GTAGAGATCGGGAGGAACACCAGTGGCGAAGGCGGCCTGCTGGGCTTTAACTGACGCTGAAGCACGAAAGCGTGG

>M00202:72:000000000-A85MG:1:1116:22967:15188;tax=Bacteria;Firmicutes;Clostridia;UNKORDER;FOTU12;GOTU299;SOTU1195;
TGGTCAATGGGCGCTAGCCTGAACCAGCCAAGTAGCGTGAAGGATGACTGCCCTATGGGTTGTAAACTTCTTTTATATGGGAATAAAGTGCAGTATGTATACTGCTTTGCA
TGTACCTTATGAATAAGGATCGGCTAACTACGTGCCAGCAGCCGCGGTAATACGTAGGAGGCAAGCGTTATCCGGAATGACTGGGCGTAAAGGGTGCGTAGGTGGTTTGGC
AAGTTAGTAGCGTAATTCCGGGGCTCAACTTCGGAACTACTACTAAAACTGCTGGGCTTGAGTGCAGGAGGGGCATATGGAATTCCTAGTGTAGCGGTGGAATGCGTAGAT
ATTAGGAGGAACACCAGTGGCGAAGGCGATATGCTGGACTGTAACTGACACTGAGGCACGAAAGCGTGG

>M00202:72:000000000-A85MG:1:1101:5139:15409;tax=Bacteria;Firmicutes;Clostridia;UNKORDER;FOTU12;GOTU327;SOTU1244;
TGGTCAATGGACGCAAGTCTGAACCAGCCATGCCGCGTGCAGGAAGACGGCTCTATGAGTTGTAAACTGCTTTTGTATTAGGGTAAACTCAGGTACGTGTACCTGACTGAA
AGTATAATACGAATAAGGATCGGCTAACTACGTGCCAGCAGCCGCGGTAATACGTAGGAGGCAAGCGTTATCCGGAATGACTGGGCGTAAAGGGTGCGTAGGTGGTTTGGC
AAGTTAGTAGCGTAATTCCGGGGCTCAACTTCGGAACTACTACTAAAACTGCTGGGCTTGAGTGCAGGAGGGGCATATGGAATTCCTAGTGTAGCGGTGGAATGCGTAGAT
ATTAGGAGGAACACCAGTGGCGAAGGCGATATGCTGGACTGTAACTGACACTGAGGCACGAAAGCGTGG
```

Figure S4. Extract from the FASTA file output produced from TIC for the Müller mice data. Every header contains a full taxonomic path up to genus, as identified by TIC. Although sOTUs, gOTUs, and fOTUs are differentiated, orders, classes and phyla are not, since there is no consensus among the scientific community on how to delineate higher taxonomic levels. Therefore, they are given as UNKPHYLUM, UNKCLASS, UNKORDER respectively.
